# Effects of compound probiotics on growth performance, immunity, antioxidant capacity and gut microbiota in weaned rabbits

**DOI:** 10.3389/fvets.2025.1714335

**Published:** 2025-11-13

**Authors:** Changchuan Ye, Menglei Shi, Jingnan Ren, Yan Zhang, Yeqiu Zhang, Yingmei Zhang, Yifei Du, Xinyi Wang, Qinghua Liu

**Affiliations:** Fujian Provincial University Engineering Research Center for Animal Breeding and Sustainable Production, College of Animal Sciences, Fujian Agriculture and Forestry University, Fuzho, China

**Keywords:** compound probiotic, weaned rabbits, growth performance, immunity, gut microbiota

## Abstract

**Introduction:**

In modern intensive animal husbandry, weaned rabbits frequently face disrupted intestinal barrier function and impaired weight gain which triggered by weaning stress. To solve this problem sustainably, innovative and effective nutritional regulation strategies should be developed. As feed additives, compound probiotics could improve feed conversion ratio and animal intestinal health and thus gain increasing recognition. Three dominant strains (*Lactobacillus plantarum* QZF, *Bacillus velezensis* BD and *Cyberlindnera fabianii* EMS) were isolated from silage and combined to form different compound probiotics. In this study, we evaluated the effect of compound probiotic on growth performance, immune function, antioxidant capacity and intestinal microbiota in weaned rabbits.

**Methods:**

A total of 64 weaned New Zealand rabbits were randomly divided into four groups. The CON group was fed a basal diet. Three treatment groups were established by supplying the following probiotics: SP Group, *L. plantarum* QZF (10^8^ CFU/d); DP1 Group, *L. plantarum* QZF (10^8^ CFU/d) and *C. fabianii* EMS (10^7^ CFU/d); DP2 Group: *L. plantarum* QZF (10^8^ CFU/d) and *B. velezensis* BD (10^8^ CFU/d).

**Results and discussion:**

Our results showed that dietary supplementation with probiotic significantly promoted growth performance (e.g., increased average daily gain and decreased feed-to-gain ratio), enhanced immune function (reflected by elevated plasmic immunoglobulin levels and reduced pro-inflammatory cytokine concentrations) and improved antioxidant capacity (manifested by higher activities of superoxide dismutase and glutathione peroxidase and lower malondialdehyde content) in weaned rabbits. Furthermore, compound probiotic supplementation influenced the composition of intestinal microflora by decreasing the abundance of *Fusobacteriota* (disease-related) and increasing the abundance of *Christensenellaceae*_R-7_group. Supplementation with compound probiotic can alter the abundance of specific gut microbiota to maintain a healthy microbial community. In conclusion, this study demonstrates that probiotics (especially compound probiotics) serve as a valuable nutritional strategy for alleviating weaning stress, optimizing growth performance and maintaining intestinal microbiota homeostasis in weaned rabbits, providing theoretical support for the application of compound probiotics in rabbit production

## 1 Introduction

As a critical transitional window when animals shift from milk-based to solid feed, the weaned period represents a pivotal phase in growth and development of animals. During this period, the maturation status of intestinal mucosal innate immune function notably exerts a decisive and long-lasting impact on the individual’s lifelong health. Enteric diseases are a major threat to the health of early life stage, particularly the weaning period (1). In particular, during the weaning period, animals are more susceptible to pathogenic bacterial invasion due to their incomplete intestinal development and underdeveloped immune systems, which can lead to a series of diseases and ultimately result in morbidity and mortality. The gastrointestinal tract serves as a vital organ for digestion, nutrient absorption and immune defense in animals. Its health status directly influences the growth, immune function and disease resistance of the animal. Enhancing gut health is considered a key strategy for improving the survival rate of neonatal animals and optimizing the economic efficiency of livestock production. A large number of studies have demonstrated that the gut microbiota plays a crucial role in maintaining metabolic functions and influencing the development and function of the immune system, thereby having significant implications for the health and growth of the host (2–4). Interventions during the early life stages of animals can facilitate the rapid establishment of a healthy gut microbiota, which in turn promotes the growth, the development of the immune system and overall health of the neonatal animals (5).

As defined by FAO and WHO, probiotics are defined as live microorganisms that confer a health benefit on the host when administered in adequate amounts. In 2014, ISAPP defined probiotics more strictly as live microbial strains (including single strains or defined human-derived consortia) that have been proven in rigorously controlled studies to be safe and effective in providing health benefits to the host when consumed in adequate amounts. Fermented foods that contain live microbes without demonstrated health effects and fecal microbiota transplants are not considered probiotics (6). In the early life stage, particularly the weaning period animals, due to their immature gastrointestinal development and underdeveloped immune systems, are particularly susceptible to pathogenic infections, which can lead to a variety of diseases. Probiotics can enhance intestinal health by improving the gut environment, regulating the balance of the intestinal microbiota, inhibiting the colonization and proliferation of pathogens and maintaining immune homeostasis, thereby promoting early-life development and alleviating post-weaning stress in young animals, such as piglets and rabbits (7, 8). Weaning is a stressful sudden transition for rabbits, including shifting from maternal milk to solid feed, separation from mother and adaptation to group housing. This change induces metabolic disturbances (e.g., lactase deficiency causing lactose malabsorption, underdeveloped pancreatic amylase impairing starch digestion) and gut microbiota dysbiosis (decreased beneficial bacteria such as *Lactobacillus* and *Bifidobacterium*, increased pathogenic bacteria such as *Escherichia coli* and *Clostridium perfringens*), ultimately reducing growth performance, increased susceptibility to specific pathogens and raising post-weaning diarrhea (PWD) incidence with elevated mortality (9, 10). Research has shown that the gut microbiota not only contributes to the maintenance of host metabolic functions but also exerts a significant influence on the development and functionality of the immune system through various mechanisms (11). Probiotics, as feed additives, have been widely utilized to enhance the growth performance of animals. Numerous studies have demonstrated that probiotics acting as live microbial feed supplements are particularly effective in managing gut microbiota, improving gut health, boosting the immunity and antioxidant capacity, enhancing colonization resistance to pathogens and optimizing feed conversion ratio (12, 13).

One of the most prominent bacteria used as probiotics belong to the genera, *Lactobacillus* (14). Recent studies have extensively documented the health benefits and underlying mechanisms of *Lactobacillus* in animals (15). *Lactobacillus* colonizes the gut epithelium (16) and exerts bactericidal effects through the production of organic acids, hydrogen peroxide and bacteriocins (17). Recent studies have demonstrated that multi-strain probiotics (also known as compound probiotics) exhibit superior efficacy compared to single-strain formulations (18). These compound probiotics have been shown to maintain intestinal mucosal homeostasis and mitigate inflammatory responses (19, 20). Besides, a combination of *Lactobacillus acidophilus* and *Bacillus subtilis* could strengthen the gut epithelial barrier and sustains immune homeostasis by modulating intestinal microbiota and metabolites (21). Supplementing diet with a compound microecological preparations in lactating female rabbits could improve milk production, immune-antioxidant status and intestinal health, thereby promoting the growth and average daily gain of their litters (22). These studies indicate that multi-strain probiotics have significant advantages in improving gut homeostasis and immune function and their combined use yields superior probiotic efficacy.

Ensiling of forages is usually recognized as a microbial-driven process (23). High-quality silage should be void of undesirable compounds or bacteria that could negatively affect animal performance, the environment, or net farm income (24). Thus, we hypothesized that high-quality silage could be a potential source of probiotic strains with excellent probiotic properties and a resulting beneficial effect on animal production. In our previous work, we successfully isolated three strains of dominant bacteria (*Lactobacillus plantarum* QZF, *Bacillus velezensis* BD and *Cyberlindnera fabianii* EMS) from silage of *Pennisetum giganteum*. In this study, these three probiotic strains were administered individually or in combination to systematically investigate the role of these probiotics on growth performance and intestinal microbiota in rabbits. This study not only proposes a nutritional regulation strategy to address weaning-induced intestinal barrier dysfunction and impaired weight gain in meat rabbit production, but also fills the gap in research on compound probiotics for weaned rabbits. Our study offered its unique value for optimizing probiotic application strategies in rabbit production.

## 2 Materials and methods

### 2.1 Moral statement

All experimental procedures and animals were approved by Fujian Agriculture and Forestry University Animal Care and Use Committee (Approval ID: PZCASFAFU24031).

### 2.2 Experimental materials

A total of 64 New Zealand White rabbits (30 d of age, with an equal number of males and females) were selected from Fujian Chunlong Agriculture and Animal Husbandry Technology Co., Ltd. (Fuzhou, China). Three probiotics, including *Lactobacillus plantarum* QZF (CGMCC No. 31389), *Bacillus velezensis* BD and *Cyberlindnera fabianii* EMS, were isolated and purified from wrapped silage of *Pennisetum giganteum*. 0.1 g of silage samples were suspended in 0.9 mL of sterile saline (0.85% *w*/*v*, pH = 7.2) and homogenized with an electrical blender for 20 min. Serial decimal dilutions were prepared in the same diluent and 0.1 mL was inoculated in triplicates by surface spreading on MRS agar. Colonies that formed clear halos were selected for further identification. These strains were all identified by 16S rDNA sequencing (*L. plantarum* and *B. velezensis*) or 18S rDNA sequencing (*C. fabianii*) and then stored at our lab.

### 2.3 Animals and housing conditions

The trial was carried out at the Rabbit Farm of Fujian Agriculture and Forestry University (Fuzhou, China) during March–May. The temperature was maintained between 22–27 °C, with relative humidity kept around 60–70%. The light management was carried out under the system of 12 L:12D (08,00–20,00 for light, 20:00–08:00 for dark). The natural ventilation was combined with the mechanical ventilation system to keep the ammonia concentration below 10 ppm. All rabbits were allocated into individual cages until slaughtering (108 days of age). Each cage was equipped with a plastic slat floor, a manual feed distribution trough and one automatic nipple drinker. Feed at 8:00 and 18:00 and provide ad libitum access to drinking water.

### 2.4 Experimental design

**Figure 1:**
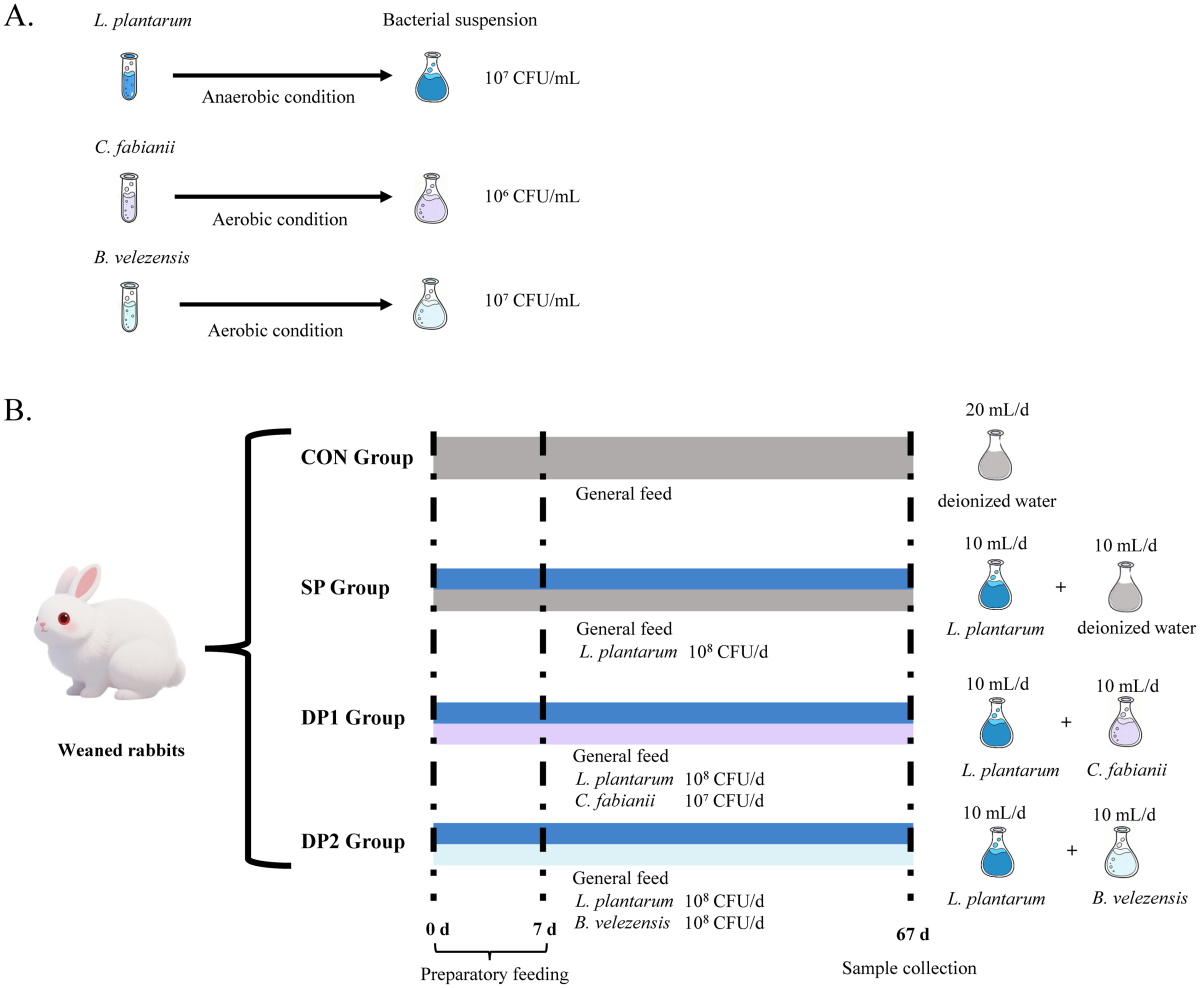
The schematic diagram of experimental design in this study. **(A)** Preparation of activated microbial suspension. **(B)** Treatment of weaned rabbits (*n* = 64).

These rabbits were randomly divided into four groups. Each group comprised four replicates and each replicate contained four rabbits (2 males and 2 females). A basal practical diet with 16% crude protein, 23% crude fiber and 260 kcal digestible energy/100 g was formulated and prepared (Table 1). As shown in Figure 1A, the three strains of probiotics were prepared to obtain 10^7^ CFU/mL (*L. plantarum* or *B. velezensis*) or 10^6^ CFU/mL (*C. fabianii*) of activated microbial suspension. The treatment of weaned rabbits was shown in Figure 1B. Rabbits were fasted for 12 h prior to gavage, which was performed between 07:00 and 08:00 each morning. A sterile stainless steel gavage needle (Biosharp, 18#, Length: 85 mm) was inserted while the animal was gently restrained in a custom fixation cradle. The operator was blinded to the treatment groups throughout the procedure. The CON group was provided with 20 mL deionized water daily per rabbit. The SP (Single-Probiotic) group was provided with 10 mL culture solution of *L. plantarum* and 10 mL deionized water daily. The DP1 (Double-Probiotic-1) group was provided with 10 mL culture solution of *L. plantarum* and 10 mL culture solution of *C. fabianii* daily. The DP2 (Double-Probiotic-2) group was provided with 10 mL culture solution of *L. plantarum* and 10 mL culture solution of *B. velezensis* daily. The experiment last for 67 consecutive days (preparatory feeding for 7 days). Immediately after the pretrial period, fecal samples from each experimental group were collected. The fecal samples of CON were used to determine fecal energy (FE) via an oxygen bomb calorimeter (RF-C7000, Ruifang Ltd., Changsha, China) for calculating the digestible energy (DE) of the feed. Throughout the trial, the animals had free access to feed and fresh water. Individual live weights were recorded twice a week to closely monitor rabbit growth and promptly detect any abnormal weight changes and health problems, while pen feed intake was measured daily. The health status of the rabbits was monitored daily. During the trial, no rabbits were excluded due to health problems.

**Table 1:** Diet composition and nutrient levels (air-dried basis).

Item	Contents
Alfalfa hay meal	28.50
Corn	26.70
Wheat bran	15.40
Rapeseed dregs	5.00
Soybean meal	8.40
Wheat middling	5.00
Unified bran	6.00
Extruded soybean	5.00
Ca(HCO3)2	0.70
Methionine	0.10
Lysine	0.20
Premix^1^	4.00
Total	100
Nutrition level^2^	
Moisture (%)	13.20
Crude fiber (%)	23.18
Crude protein (%)	16.13
Ether extract (%)	0.70
Calcium (%)	1.41
Phosphorus (%)	0.79
GE^3^ (kcal/100 g)	380.39
DE^4^ (kcal/100 g)	263.27
P:E^5^ (mg protein/MJ DE)	61.46

^1^ The premix provided Cu 5 mg; Fe 100 mg; Zn 50 mg; Mn 30 mg; Mg 150 mg; I 1 mg (inorganic); Se 0.1 mg; VA 8000 IU; VD 800 IU; VE 50 mg; VB1 2 mg; VB2 6 mg; VB6 2 mg; VB12 0.01 mg; folic acid 1 mg; pantothenic acid 20 mg per kg of diet. ^2^ Moisture, Crude protein (CP), crude fiber (CF), ether extract (EE), calcium (Ca) and phosphorus (P) were measured and recorded. CP was determined by the Kjeldahl method; CF were measured using the filter bag technique; Ca was determined by the ethylenediaminetetraacetic acid (EDTA) complexometric titration method; P was measured by spectrophotometry at a wavelength of 400 nm with the molybdenum blue reaction. ^3^ Gross energy, was measured by an oxygen bomb calorimeter (RF-C7000, Ruifang Co., Ltd., Changsha, China). ^4^ DE, Digestible energy = GE - FE. FE (Fecal energy) was measured by an oxygen bomb calorimeter (RF-C7000, Ruifang Ltd., China). ^5^ P:E, Protein to energy ratio = (CP% × 1,000)/DE (kcal/100 g).

### 2.5 Growth performance and slaughtering

Feed intake was recorded daily. At 108 days of age, after a fasting period of 4 h, all rabbits in the trial were weighed at the experimental farm to determine the final body weight. Then, 16 rabbits (1 rabbits per replicate, two males and two females per diet), representative of the corresponding experimental groups in terms of average rabbit live weight and SD, were individually selected to minimize the confounding effect of sex on the experimental results. These rabbits were weighed, stunned using electro anesthesia and slaughtered by jugulation.

### 2.6 Sample collection

After slaughtering, blood was immediately taken from the heart to obtain samples with a more consistent mixture of systemic blood. Once collected, the blood was placed into lithium-heparin tubes. The plasma was directly obtained by centrifugation (Thermo Scientific SL, 16R, Thermo Fisher Scientific, Waltham, MA, USA) at 4000 g for 10 min, 4 °C and stored at −20 °C.

The dorsal muscle was selected as sample for analysis. The pH of muscle was measured by a hand-held pH meter (PHSJ-3F, Shanghai, China). Drip loss was determined by hanging a section from a 5 cm × 3 cm × 2 cm muscle sample in a sealed plastic bag for 24 h at 4 °C. Shear force was determined by cutting a 3 cm × 1 cm × 1 cm piece of muscle and testing it with a digital muscle tenderness tester (C-LM3B, Tenovo, Beijing, China).

The intestine (including duodenum, jejunum, ileum and colon) were excised and cleaned with saline. The mucosa was scraped off and these samples were stored at −80 °C. Inflammatory factors (including IL-1α, IL-2 and sIgA) by ELISA kits (Shanghai Liquid Quality Testing Technology Co., Ltd., Shanghai, China) according to the instructions of manufacturer. Duodenal, jejunal and ileal segments were fixed in 4% paraformaldehyde for histological examination. Then villus height (VH), crypt depth (CD), VH/CD ratio and goblet cell (GC) were measured.

Parts of the duodenum and cecum were finely homogenized in saline using a homogenizer (FSH-2A, Japid Co., Ltd., Qingdao, China) and centrifuged at 8000 rpm for 5 min, 4 °C to obtain the supernatants. The activities of amylase, cellulase, lipase and trypsin were measured by commercial assay kits (Shanghai Liquid Quality Testing Technology Co., Ltd., Shanghai, China) according to the instructions of manufacturer. Samples of cecal contents were aseptically collected in 2-ml Eppendorf tubes and stored at −80 °C until analysis.

### 2.7 Chemical and biochemical analysis

The plasmic biochemical parameters were determined using an automatic biochemical analysis system (BS−200, Mindray Biomedical Electronics Co., Ltd., Shenzhen, China). A comprehensive set of biochemical parameters were measured, including total protein (TP), triglyceride (TG), high-density lipoprotein cholesterol (HDL−C), total cholesterol (TC) and low-density lipoprotein cholesterol (LDL−C).

Free fatty acid (FFA) levels were tested by commercial test kit (Nanjing Jiancheng Technology Co., Ltd., Nanjing, China) according to the instructions of manufacturer. Total antioxidant capacity (T-AOC), plasmic superoxide dismutase (SOD) and plasmic malondialdehyde (MDA) content were measured by using the commercial assay kits (Shanghai Liquid Quality Testing Technology Co., Ltd., Shanghai, China) according to the instructions of manufacturer.

IgA, IgG, IgM, IL-1α, IL-2 and sIgA were measured by ELISA kits (Shanghai Liquid Quality Testing Technology Co., Ltd., Shanghai, China) according to the instructions of manufacturer.

### 2.8 Cecal fermentation

The pH values of the cecum were measured using a pH meter (PHSJ-3F, Leici, Shanghai, China).

The contents of volatile fatty acids (VFA), including acetic acid, propionic acid, butyric acid, isobutyric acid and isovaleric acid, were measured by gas chromatograph system (GC 2010-FID, Shimadzu, Kyoto, Japan). The gas chromatograph system was equipped with a column (KB-FFAP, Kromat, Delran, NJ, USA) and a flame ionization detector was operated at 250 °C. A 0.81 mL/min mobile phase using N_2_ was applied to the column. The column was operated at 80 °C.

Ammoniacal nitrogen (NH_3_−N) content was measured using phenol-sodium hypochlorite colorimetry, following the Broderick and Kan method (25).

### 2.9 Cecal microbiota analysis

The microbial flora analysis was detected by 16SrDNA high-throughput sequencing technology as follows steps. First, fresh cecal contents were collected and store at −80 °C until testing. Then, the total DNA of microorganisms was extracted using the QIAamp DNA Stool Mini Kit (QIAGEN, Germany) according to the instructions of manufacturer.

The 16S rRNA gene of the samples was amplified and sequenced using the Miseq PE3000 high-throughput sequencing platform of Illumina. The amplification and sequencing primers were the universal primers 27F and 1492R. The nucleotide sequence of 27F is 5′-AGAGTTTGATCCTGGCTCA-3′; The nucleotide sequence of 1492R is 5′-GGTTACCTTGTTACGACTT-3′. Immediately, the Miseq library was established and sequenced on the machine. The sequencing data were demultiplexed into individual samples based on barcode sequences using QIIME (v1.8.0). Raw reads were filtered and merged with PEAR (v0.9.6); reads with a quality score <20, ambiguous bases, or primer mismatches were discarded. During merging, the minimum overlap was set to 10 bp and the mismatch rate to 0.1. After merging, VSEARCH (v2.7.1) was employed to remove sequences shorter than 230 bp and chimeric sequences were identified and eliminated by the UCHIME algorithm against the Gold Database. High-quality sequences were then clustered into operational taxonomic units (OTUs) using the UPARSE algorithm in VSEARCH (v2.7.1) with a 97% similarity threshold. Representative OTU sequences were taxonomically assigned by BLAST against the SILVA 138 database with an e-value cutoff of 1e-5 for OTU clustering of valid sequences. Then data analysis processes such as species annotation, diversity analysis, species difference comparison and association analysis were carried out. The intestinal microbiota section is entrusted to (Beijing Aoweisen Gene Technology Co., LTD.) for testing the relative abundance of intestinal flora, etc. The accession number for sequencing data is PRJNA1345346 in Entrez.

### 2.10 Statistics analysis

A completely randomized experimental design was employed in this study. All data were analyzed using IBM SPSS Statistics software (version 25.0). Data were first subjected to normality testing using the Shapiro–Wilk test. For datasets conforming to normal distribution, Bartlett’s test was employed to assess homogeneity of variance. The results of Shapiro–Wilk test and Bartlett’s test were shown in Supplementary Table S1. When homogeneity of variance was confirmed, one-way analysis of variance (ANOVA) was performed; otherwise (heterogeneous variance or non-normal distribution), the non-parametric Kruskal–Wallis test was applied. Unless otherwise stated, all data met the assumption of homogeneity of variance. The CON group was set as the control. When the ANOVA or Kruskal–Wallis test indicated significant differences among groups, Tukey’s multiple range test was applied with a Bonferroni correction to identify which specific pairs of groups differed significantly. The results are presented as the mean ± standard error (SEM). The 16S rDNA sequencing data were analyzed on the free online platform of Omicsmart tools^1^ and the Kruskal–Wallis test was employed for analysis of significant differences. In this study, statistical significance was set at *p* < 0.05 and trend identified at 0.05 ≤ *p* < 0.10.

## 3 Results

### 3.1 Growth performance and meat quality

Growth performance and Meat quality were shown in Table 2. The three groups treated with probiotics (SP, DP1 and DP2) significantly increased final body weight (BW), average daily feed intake (ADFI) and average daily gain (ADG) in weaned rabbits compared to the CON group (*p <* 0.01). The feed-to-gain ratio (F/G) was significantly reduced in the SP and DP1 groups compared to the CON group. Furthermore, we found that the shear force of the DP2 group showed a decreasing trend compared with the control group (*p =* 0.054).

**Table 2:** Effects of probiotics on the growth performance of weaned rabbits.

Items	Groups				*p*-value
	CON	SP	DP1	DP2	
Growth performance					
IBW (g) *	821 ± 28	775 ± 23	794 ± 28	831 ± 28	0.504
FBW (g)	2,283 ± 53^b^	2,578 ± 62^a^	2,550 ± 45^a^	2,493 ± 42^a^	<0.001
ADFI (g)	126 ± 3^b^	142 ± 3^a^	140 ± 2^a^	137 ± 2^a^	<0.001
ADG (g)	24.36 ± 1.16^b^	30.05 ± 1.02^a^	29.27 ± 0.92^a^	27.71 ± 0.66^a^	<0.001
F/G *	5.25 ± 0.14^a^	4.75 ± 0.07^b^	4.83 ± 0.10^b^	4.97 ± 0.08^ab^	0.027
Meat quality					
pH24h	6.46 ± 0.08	6.47 ± 0.05	6.58 ± 0.06	6.45 ± 0.01	0.397
Drip loss (%)	3.29 ± 0.91	2.32 ± 0.84	1.37 ± 0.44	2.42 ± 0.43	0.321
Shear force (N)	36.05 ± 5.02	28.75 ± 5.45	36.20 ± 1.93	20.96 ± 1.75	0.054

IBW, Initial body weight; FBW, final body weight; ADFI, average daily feed intake; ADG, average daily gain; F/G, feed/gain ratio. The results are presented as the mean ± SEM. For growth performance, *n* = 16. For meat quality, *n* = 4. * Indicates that the data do not meet normality or the variances are not homogeneous, thus the Kruskal–Wallis test was used. For others, ANOVA was used. Different letters indicate significant differences (*p <* 0.05).

### 3.2 Changes in plasmic metabolites and antioxidant capacity

As shown in Table 3, supplementing probiotic with *L. plantarum* alone (SP group) significantly increased the BUN level in the plasmic of weaned rabbits compared to the CON group (*p <* 0.05). We also found that the DP1 group, which was supplemented with *L. plantarum* and *C. fabianii*, tended to increase plasma HDL-C levels compared with the CON group (*p* = 0.075).

**Table 3:** Effect of probiotics on plasmic biochemical indexes of weaned rabbits.

Items	Groups				*p*-Value
	CON	SP	DP1	DP2	
TP (g/L)	53.90 ± 0.93	54.03 ± 1.52	54.00 ± 2.63	54.70 ± 2.36	0.991
ALB (g/L) *	35.57 ± 0.64	36.63 ± 0.32	37.47 ± 1.68	35.40 ± 0.75	0.576
TG (mmol/L)	0.44 ± 0.07	0.59 ± 0.05	0.60 ± 0.06	0.55 ± 0.17	0.653
TC (mmol/L)	1.17 ± 0.21	1.55 ± 0.33	1.69 ± 0.14	1.70 ± 0.37	0.532
GLU (mmol/L)	6.07 ± 0.23	6.64 ± 0.34	7.00 ± 0.46	6.89 ± 0.24	0.266
BUN (μg/mL)	24.02 ± 3.02^b^	35.44 ± 2.81^a^	25.59 ± 3.12^ab^	19.15 ± 3.54^b^	0.035
HDL-C (mmol/L) *	0.74 ± 0.07	0.68 ± 0.02	0.98 ± 0.07	0.82 ± 0.07	0.075
LDL-C (mmol/L)	0.14 ± 0.02	0.35 ± 0.10	0.50 ± 0.09	0.65 ± 0.20	0.011

TP: Total protein; ALB: Albumin; TG: Triglyceride; TC: Total cholesterol; GLU: Glucose; BUN: Urea nitrogen; HDL-C: High-density lipoprotein; LDL-C: Low-density lipoprotein. The results are presented as the mean ± SEM. *n* = 3. * Indicates that the data do not meet normality or the variances are not homogeneous, thus the Kruskal–Wallis test was used. For others, ANOVA was used. Different letters indicate significant differences (*p <* 0.05).

**Figure 2:**
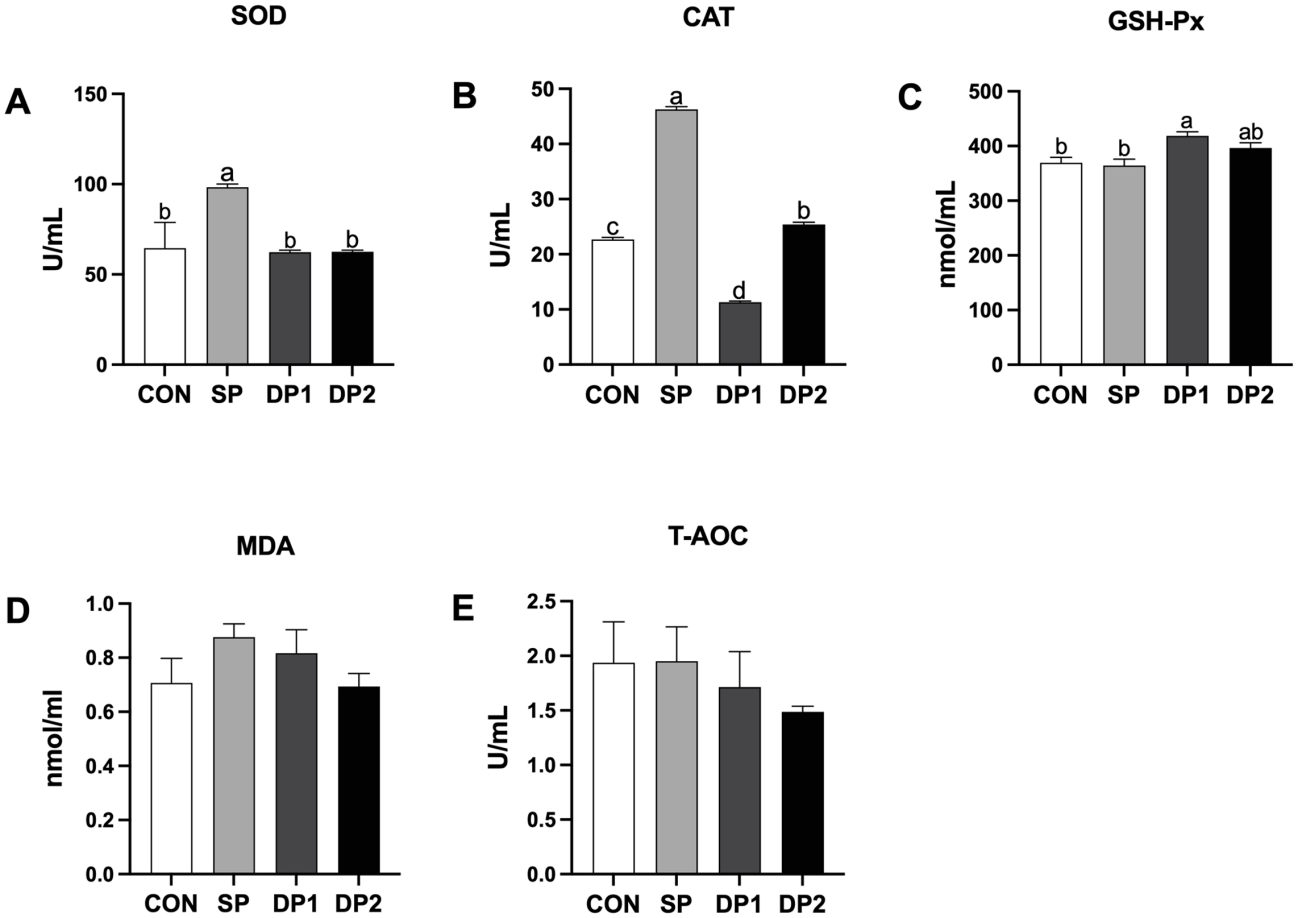
The plasmic antioxidant capacity of weaned rabbits following probiotics supplementation. **(A)** SOD: Superoxide dismutase; **(B)** CAT: Catalase; **(C)** GSH-Px: Glutathione peroxidase; **(D)** MDA: Malondialdehyde; **(E)** T-AOC: Total antioxidant capacity. The error bar indicates SEM. Different letters indicate significant differences (*p <* 0.05). *n* = 3. These data are from Supplementary Table S2.

As shown in Figure 2, the DP1 group showed a significant higher activity of SOD and CAT compared with the CON group. Specifically, the SOD activity in the DP1 and DP2 groups showed no significant difference compared to the control group. The supplementation of compound probiotic with *B. velezensis* and *C. fabianii* significantly inhibited CAT activity. Although the supplementation of compound probiotic with *B. velezensis* and *L. plantarum* increased the activity of CAT, the effect was significantly less compared to the separate supplementation of *L. plantarum*. The combined supplementation with *L. plantarum* QZF and *C. fabianii* EMS (DP1 group) significantly elevated glutathione peroxidase (GSH-Px) activity. No statistically significant differences were observed in the level of MDA and T-AOC among these groups (*p* > 0.05).

### 3.3 Changes in immunity

**Figure 3:**
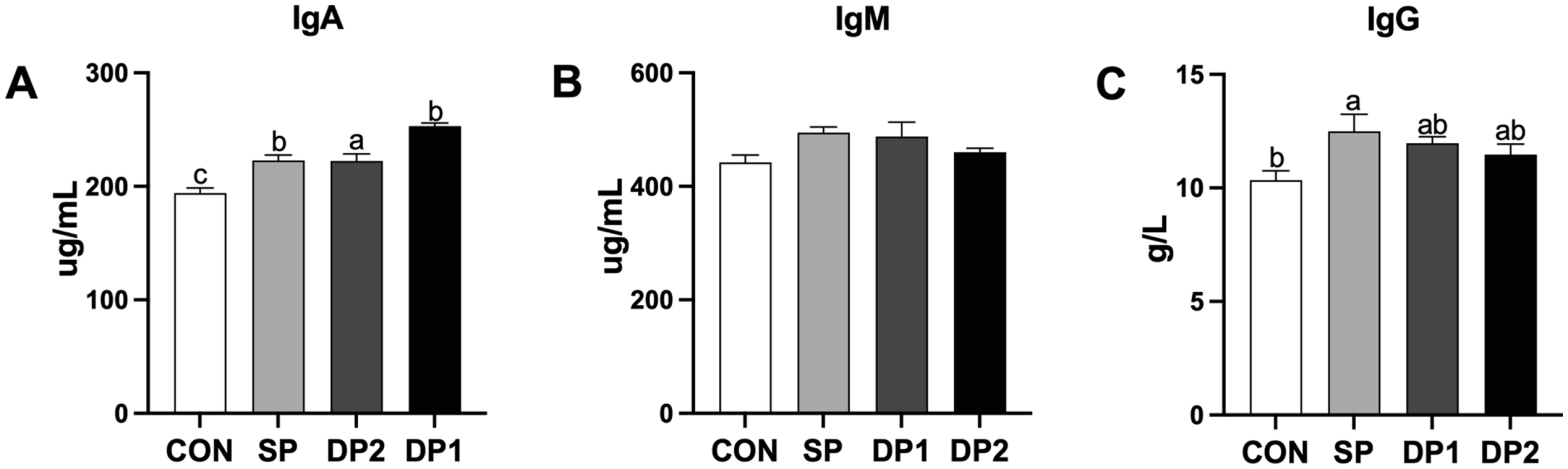
The plasmic immune factor levels of weaned rabbits following probiotics supplementation. **(A)** IgG: Immunoglobulin G; **(B)** IgA: Immunoglobulin A; **(C)** IgM: Immunoglobulin M. The error bar represents the standard error (SEM). Different letters indicate significant differences (*p <* 0.05). *n* = 3. These data are from Supplementary Table S3.

According to Figure 3, compared with the CON group, all groups that supplemented with probiotics (SP, DP1 and DP2 groups) have a significant higher level of IgA in the plasma (*p <* 0.05). Single-strain probiotic supplement with *L. plantarum* QZF could significantly increase the level of IgG in the plasma (*p* < 0.05), compared to CON group. No significant differences were observed in plasmic IgM level among these groups.

**Figure 4:**
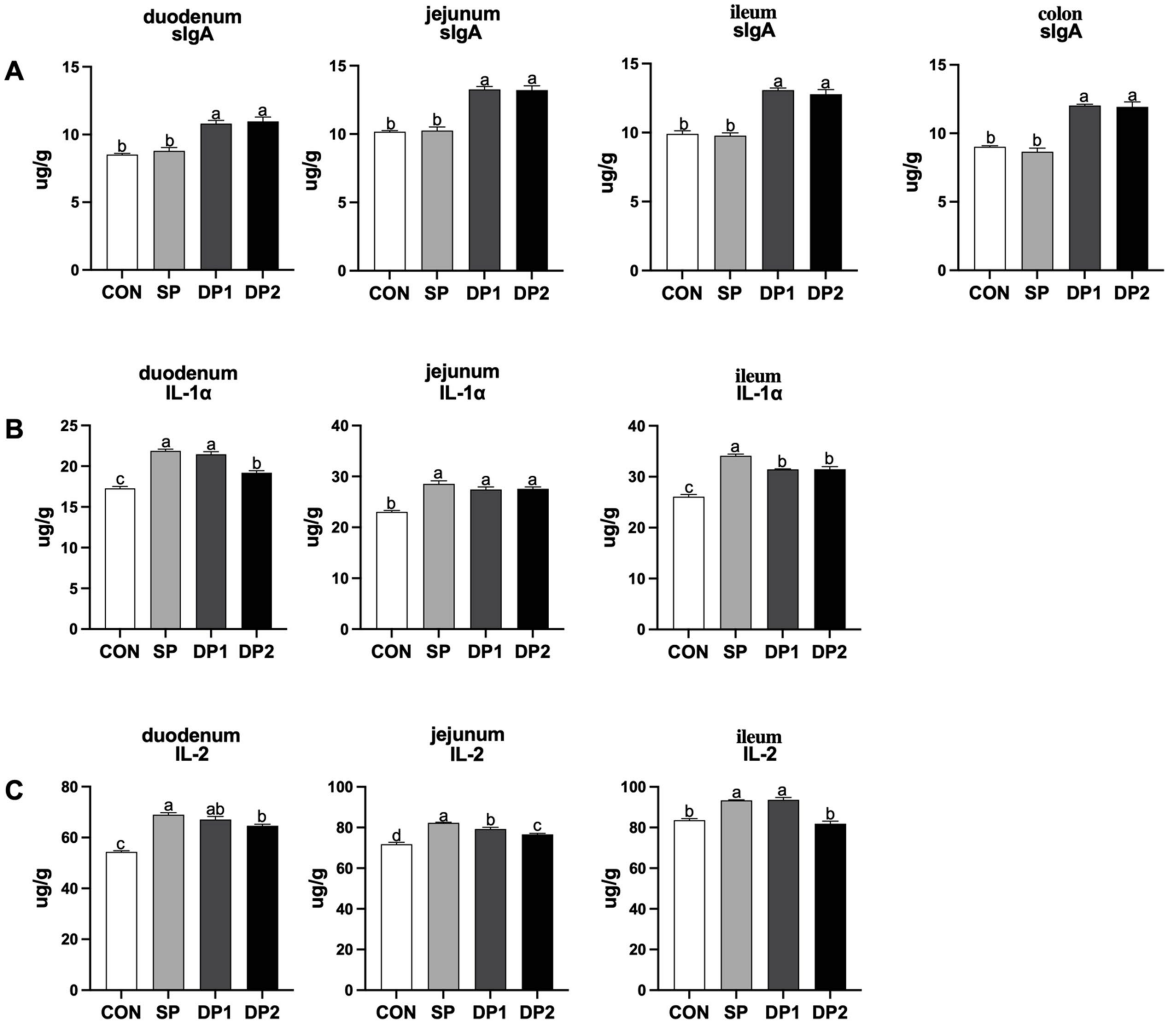
The intestinal immune factor levels of weaned rabbits following probiotics supplementation. **(A)** sIgA: Secretory immunoglobulin A; **(B)** IL-1α: Interleukin 1α; **(C)** IL-2: Interleukin 2. The error bar indicates standard error (SEM). Different letters indicate significant differences (*p <* 0.05). *n* = 3. These data are from Supplementary Table S4.

The data on the intestinal mucosal immunity are shown in Figure 4. As shown in Figure 4A, the addition of compound probiotics (DP1 and DP2 group) could significantly increase the levels of sIgA in the intestine (including duodenum, jejunum, ileum and colon). According to Figure 4B, compared with the CON group, all groups that supplemented with probiotics (SP, DP1 and DP2 groups) have a significant higher level of IL-1α in duodenum, jejunum and ileum (*p <* 0.05). Notably, DP2 showed a significantly lower level than SP and DP1 but a significantly higher level than CON for IL-1α in duodenum. We also observed that supplemented with probiotics (SP, DP1 and DP2 groups) could significantly increase the level of IL-2 in duodenum and jejunum. In ileum, the level of IL-2 significantly increased in SP and DP1 groups, compared with CON group (*p <* 0.05).

### 3.4 Changes in intestinal morphology and enzyme level

**Figure 5:**
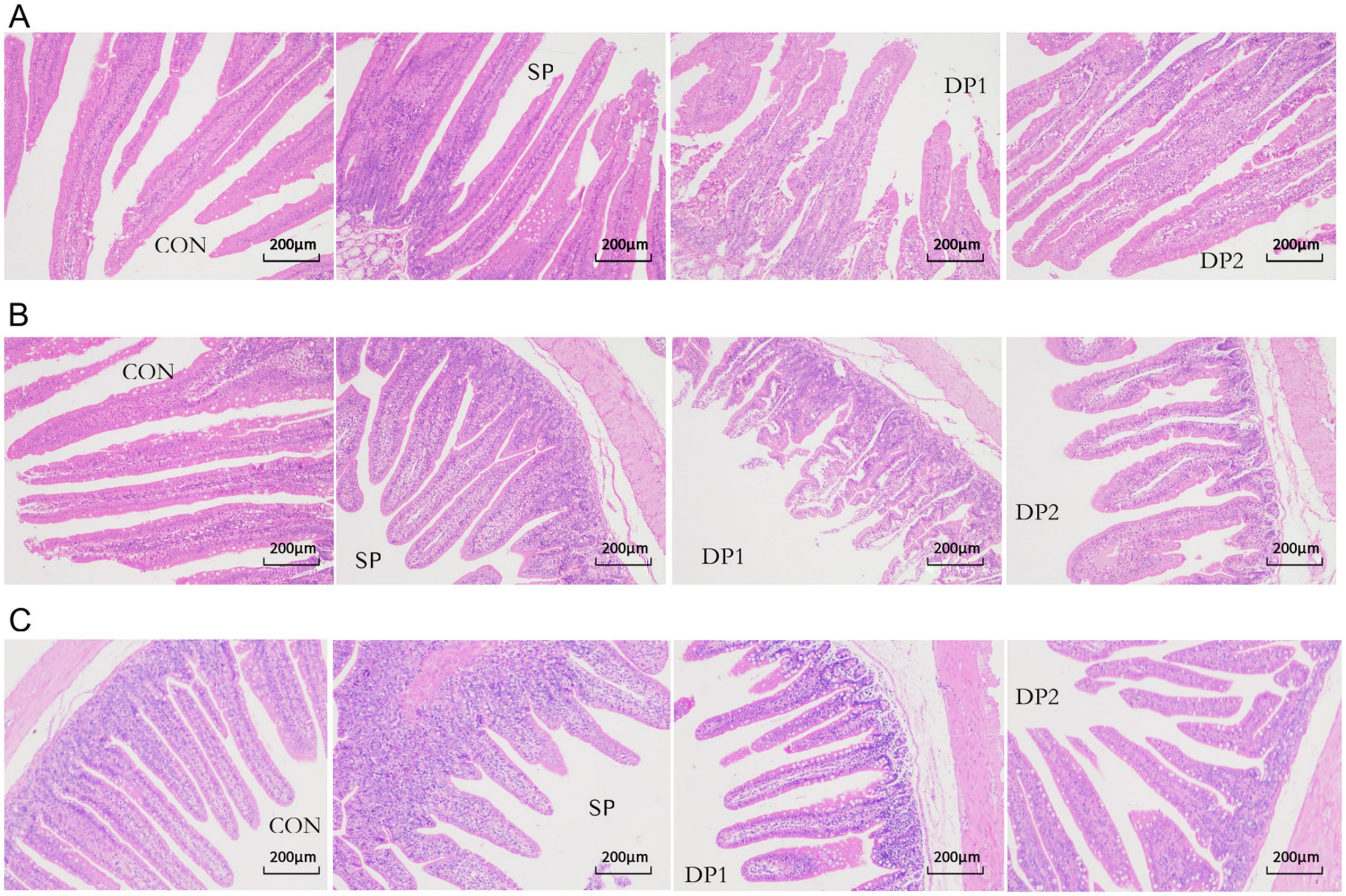
Microscopic images of the HE slide (100×) of intestine. Microscopic images of the HE slide (100×) of duodenum **(A)**, jejunum **(B)**, and ileum **(C)** from the CON, SP, DP1 and DP2 group. The histological analyses were shown in Table 4.

The microscopic images of the hematoxylin–eosin (HE) slide were shown in Figure 5 while the histological analyses are summarized in Table 4. Compared with the CON group, the supplement of compound probiotic (DP1 and DP2 groups) significantly increased crypt depth (CD) in the duodenum (*p* < 0.05), while villus height (VH) significantly increased in DP2 group (*p <* 0.01). In jejunum, we found that all groups that treated with probiotics (SP, DP1 and DP2 groups) showed a significant lower villus height and crypt depth, compared with CON group. A significant decrease of goblet cell density in duodenum was observed in DP1 group. In jejunum, all groups that treated with probiotics (SP, DP1 and DP2 groups) showed significant lower VH and CD (*p <* 0.01), compared with CON group. The V/C ratio in jejunum also significantly decreased in SP1 and DP2 groups. We also found that probiotics treated groups (SP, DP1 and DP2 groups) showed a significant lower CD in ileum, compared with CON group (*p* < 0.05). Notably, V/C ratio and goblet cell density in DP2 groups was significantly higher (*p <* 0.01) than that in CON group in ileum. The above results indicate that the supplementation of compound probiotics (*L. plantarum*, *B. velezensis* or *C. fabianii*) has a certain ameliorating effect on the intestinal morphology of the duodenum in rabbits.

**Table 4:** Effect of probiotics on gut morphometry and morphology of weaned rabbits.

Items	Groups				*p*-value
	CON	SP	DP1	DP2	
Duodenum					
Villus height (*μ*m) *	938 ± 57^b^	926 ± 24^b^	1,007 ± 40^ab^	1,089 ± 35^a^	0.044
Crypt depth (μm)	96.15 ± 4.37^b^	91.05 ± 3.29^b^	113.47 ± 6.14^a^	113.75 ± 5.19^a^	0.002
V/C	10.11 ± 0.77	10.41 ± 0.55	9.44 ± 0.84	9.97 ± 0.72	0.821
Goblet cell density (cells/mm)	12.53 ± 0.74^a^	13.47 ± 0.77^a^	9.73 ± 0.35^b^	13.47 ± 0.93^a^	0.021
Jejunum					
Villus height (μm) *	944 ± 85^a^	591 ± 17^b^	549 ± 22^b^	618 ± 23^b^	0.005
Crypt depth (μm)	120.09 ± 6.31^a^	103.76 ± 4.86^b^	86.05 ± 5.38^c^	105.81 ± 2.78^b^	<0.001
V/C *	7.99 ± 0.75	5.80 ± 0.20	6.79 ± 0.58	5.88 ± 0.26	0.176
Goblet cell density (cells/mm) *	12.13 ± 2.37	7.47 ± 0.97	8.40 ± 0.69	8.13 ± 0.18	0.292
Ileum					
Villus height (μm) *	618 ± 16	536 ± 38	598 ± 66	636 ± 26	0.228
Crypt depth (μm) *	150.35 ± 7.88^a^	124.39 ± 4.93^b^	122.09 ± 6.25^b^	117.92 ± 13.50^b^	0.004
V/C *	4.30 ± 0.30^b^	4.37 ± 0.32^b^	5.25 ± 0.77^ab^	5.95 ± 0.43^a^	0.033
Goblet cell density (cells/mm) *	17.67 ± 1.12^bc^	17.07 ± 0.33c	19.80 ± 0.81^ab^	22.13 ± 0.47^a^	0.005

V/C: Villus height/Crypt depth ratio. The results are presented as the mean ± SEM. *n* = 5. * Indicates that the data do not meet normality or the variances are not homogeneous, thus the Kruskal–Wallis test was used. For others, ANOVA was used. Different letters indicate significant differences (*p <* 0.05).

As shown in Table 5, compared with the CON group, all groups treated with probiotics (SP, DP1 and DP2 groups) showed significantly levels of amylase in the duodenum (*p <* 0.01). In cecum, the level of amylase significantly increased in SP group while significantly decreased in DP1 group (*p <* 0.01).

**Table 5:** Effect of probiotics on intestinal enzyme level of weaned rabbits.

Items	Groups				*p*-value
	CON	SP	DP1	DP2	
Duodenum					
Amylase (nmol/L) *	404 ± 4^c^	581 ± 12^a^	550 ± 9^b^	567 ± 7^ab^	0.038
Cellulase (ng/mL)	12.95 ± 0.92	18.16 ± 4.43	14.02 ± 2.98	16.37 ± 1.81	0.592
Lipase (mg/mL)	221 ± 13	225 ± 6	260 ± 25	218 ± 16	0.303
Trypsin (μ/g)	14.10 ± 1.70	12.11 ± 1.24	12.19 ± 3.48	11.56 ± 1.05	0.838
Cecum					
Amylase (nmol/L)	202 ± 1^b^	218 ± 1^a^	182 ± 2^c^	201 ± 1^b^	<0.001
Cellulase (ng/mL) *	20.90 ± 0.86	27.25 ± 7.00	25.31 ± 6.79	14.34 ± 1.58	0.187
Lipase (mg/mL)	240 ± 10	242 ± 10	225 ± 12	239 ± 5	0.619
Trypsin (μ/g)	12.43 ± 0.20	10.22 ± 1.43	10.69 ± 2.14	14.13 ± 2.40	0.432

The results are presented as the mean ± SEM. *n* = 3. * Indicates that the data do not meet normality or the variances are not homogeneous, thus the Kruskal–Wallis test was used. For others, ANOVA was used. Different letters indicate significant differences (*p <* 0.05).

### 3.5 Cecal fermentation

As shown in Table 6, the pH of cecal content significantly decreased when treated with *L. plantarum* separately (*p <* 0.01). The content of NH_3_-N significantly increased in probiotic treated groups (SP, DP1 and DP2 groups). We also found that the content of isovaleric acid significantly decreased in DP1 and DP2 groups (*p <* 0.05).

**Table 6:** Effect of probiotics on cecal fermentation of weaned rabbits.

Items	Groups				*p-*value
	CON	SP	DP1	DP2	
pH *	6.61 ± 0.04^a^	6.37 ± 0.04^b^	6.69 ± 0.08^a^	6.62 ± 0.02^a^	<0.001
NH_3_-N(μg/mL)	1.03 ± 0.01^c^	1.25 ± 0.02^b^	1.23 ± 0.01^b^	1.34 ± 0.03^a^	<0.001
Acetic acid (μg/g)	292 ± 15	415 ± 65	463 ± 139	443 ± 62	0.504
Propionic acid (μg/g) *	100 ± 5	152 ± 34	139 ± 36	119 ± 3	0.400
Butyric acid (μg/g)	161 ± 16	248 ± 59	256 ± 105	260 ± 67	0.714
Isobutyric acid (μg/g)	19.36 ± 2.03	18.94 ± 1.28	13.74 ± 2.76	13.26 ± 1.10	0.101
Isovaleric acid (μg/g)	16.99 ± 1.81^a^	16.59 ± 0.83^a^	9.99 ± 1.49^b^	10.47 ± 0.83^b^	0.008

The results are presented as the mean ± SEM. For pH: *n* = 4. For others, *n* = 3. * Indicates that the data do not meet normality or the variances are not homogeneous, thus the Kruskal–Wallis test was used. For others, ANOVA was used. Different letters indicate significant differences (*p <* 0.05).

### 3.6 Intestinal microbiota community

**Figure 6:**
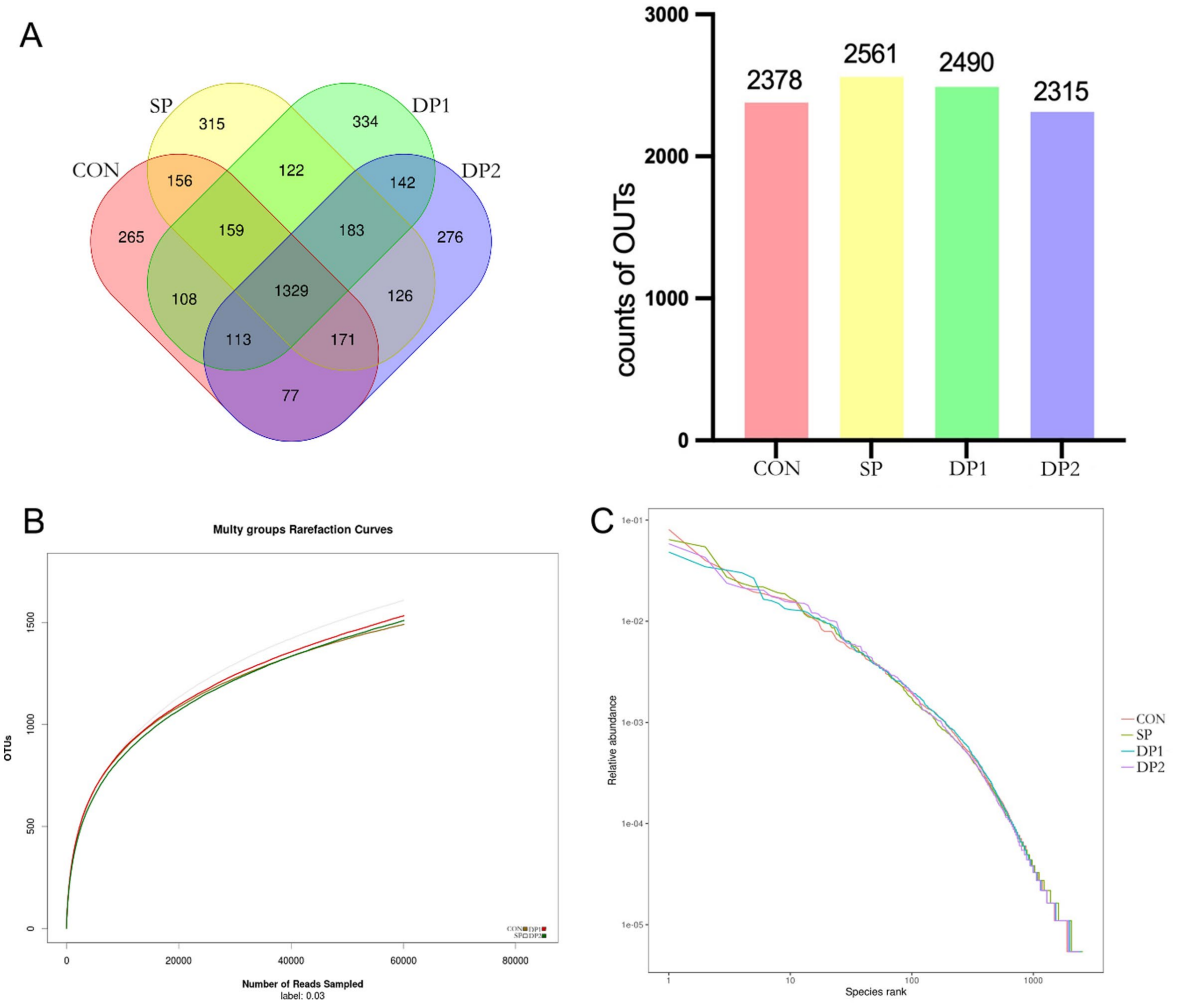
The OTU Venn diagram, dilution curve and rank-abundance curve of cecal microbiota. **(A)** The OTU Venn diagram, **(B)** Dilution curve, **(C)** Rank-abundance curve. *n* = 3.

To investigate the changes in gut microbiota composition and structure between these groups of weaned rabbits, 16S rRNA gene amplicon sequencing was performed on cecal contents. As illustrated in the Venn diagram (Figure 6A), a total of 9,744 OTUs were identified across all four groups (CON: 2378, SP: 2561, DP1: 2490, DP2: 2315). Among these, only 1,190 OTUs (CON: 265, SP: 315, DP1: 334, DP2: 276) were unique to each group. This substantial overlap suggests a core microbiome structure conserved across treatments. The rarefaction curve can be used to judge whether the depth of sequencing is sufficient. The dilution curve of each group was relatively stable (shown in Figure 6B). These results indicated that the sequencing data amount of cecal microflora of weaned rabbits in this experiment was enough. The sequencing columns were sufficient to cover the composition of microbial community species, which could truly reflect the proportional relationship among various species in the community. The extended horizontal axis span observed in the rank-abundance curve (shown in Figure 6C) demonstrated high species richness in all groups. While vertical axis uniformity across groups suggested comparable evenness distributions. All the curves showed no separation and were slightly steep. These results of rank-abundance curves indicated that the species distribution are unevenly, with a few species dominating.

The Alpha-diversity analysis was shown in Table 7, all groups exhibited 99% sequence coverage, confirming that the sequenced libraries effectively represented the bacterial diversity within cecal contents. There were no significant differences in Chao1, species, whole and Shannon indices across all groups (*p* > 0.05).

**Table 7:** Effect of probiotics on cecal microbial alpha diversity of weaned rabbits.

Items	Groups				*p*-value
	CON	SP	DP1	DP2	
Coverage	0.99	0.99	0.99	0.99	/
Chao1	1893 ± 35	1985 ± 47	1852 ± 158	1791 ± 63	0.524
Species	1,537 ± 37	1,636 ± 10	1,547 ± 89	1,499 ± 68	0.463
Whole	86.80 ± 0.96	91.31 ± 0.72	85.25 ± 3.77	83.00 ± 2.81	0.180
Shannon	7.53 ± 0.17	7.45 ± 0.15	7.60 ± 0.11	7.45 ± 0.19	0.887

The results are presented as the mean ± SEM. *n* = 3. The Kruskal–Wallis test was used. Different letters indicate significant differences (*p <* 0.05).

**Figure 7:**
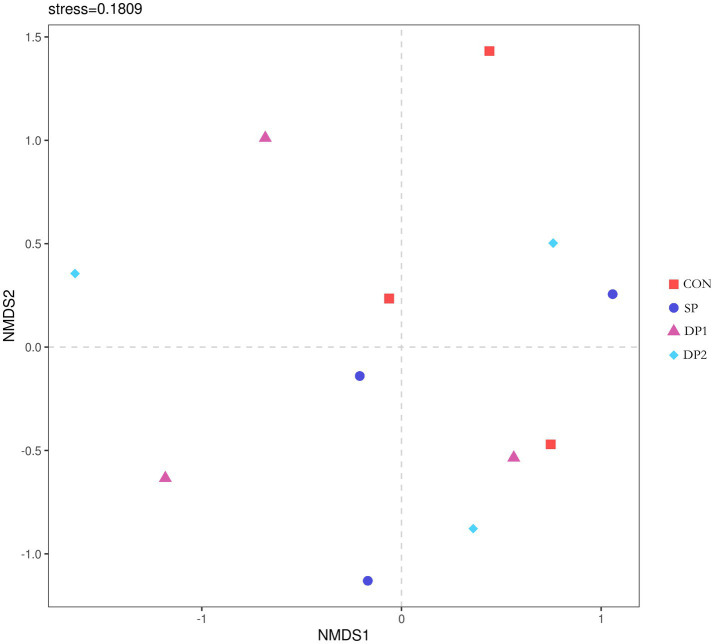
The NMDS analysis diagram. The Stress value is a measure of distortion. It is generally considered that when Stress is less than 0.2, the results of NMDS analysis are reasonably reliable. *n* = 3.

The Beta-diversity analysis was shown in Figure 7. NMDS analysis was performed on bacterial communities based on the Bray-Curtis distance algorithm. The stress value was 0.1809 (less than 0.2), indicating a good fit of the model. The points of these groups did not separate from each other, indicating that the bacterial community structures of these groups were similar.

**Figure 8:**
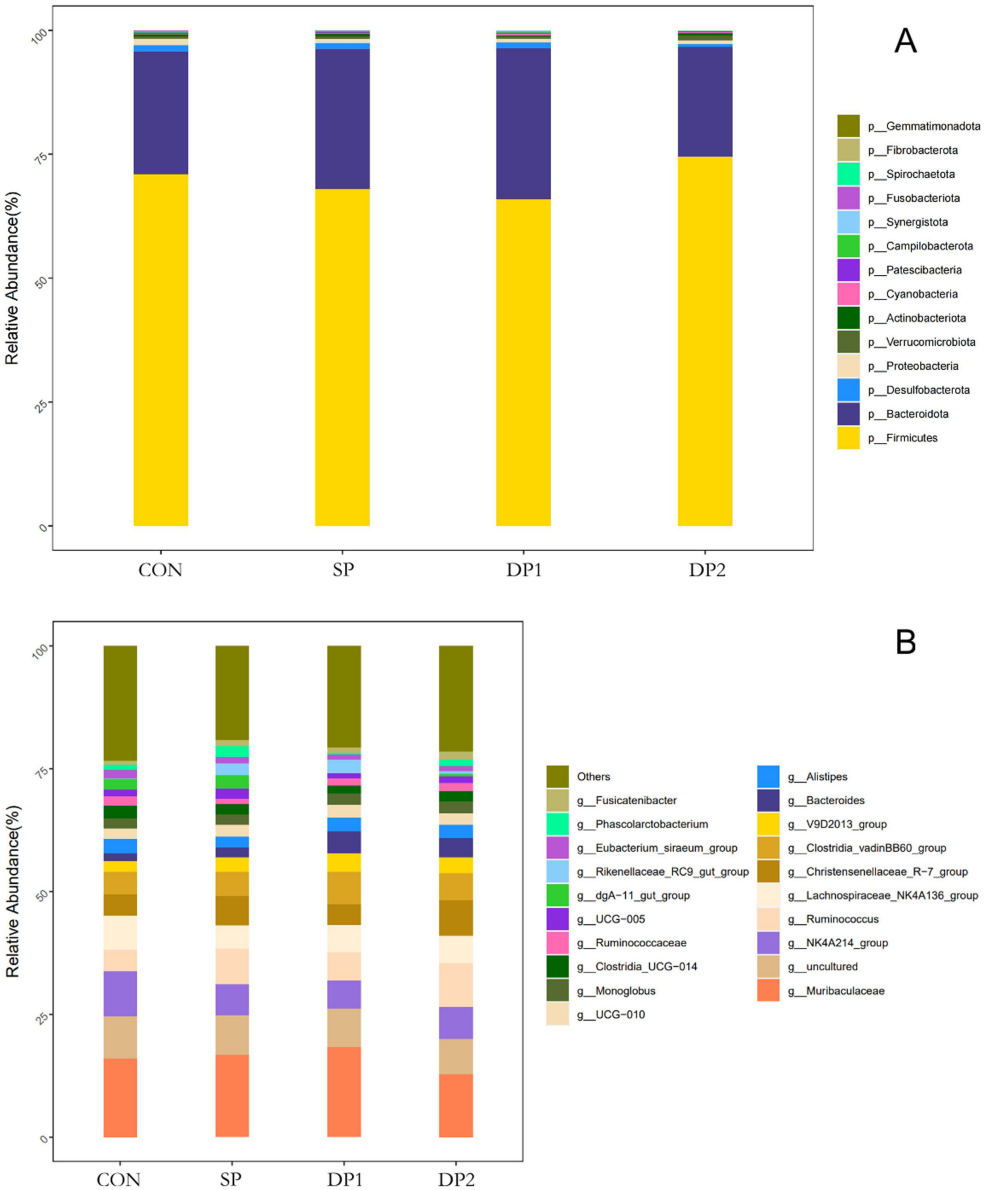
The relative abundance of OTUs about the cecal microflora. **(A)** Cecal microbiota richness and diversity at phylum level; **(B)** Cecal microbiota richness and diversity at genus level. n = 3.

These findings collectively indicate that dietary supplementation with probiotics such as *L. plantarum*, *B. velezensis* or *C. fabianii* did not have significant impact on the Alpha-diversity, Beta-diversity and microbial community structure of cecal microbiota in healthy weaned rabbits. As shown in Figure 8, *Firmicutes*, *Bacteroidetes*, *Proteobacteria* and *Desulfobacterota* predominated the cecal microbiota of each group at the phylum level. At the genus level, *Muribaculaceae*, NK4A214_group, uncultured, *Lachnospiraceae*_NK4A136_group and *Christensenellaceae*_R-7_group were the primary genera of cecal microbial community. The statistical analyses are summarized in Table 8. Kruskal-Wallis analysis showed that compound probiotic supplementation (DP1 and DP2 groups) significantly decreased the abundance of *Fusobacteriota* at the phylum level. At genus level, the abundance of NK4A214_group significantly decreased in probiotic treated groups (SP, DP1 and DP2 groups). The abundance of *Christensenellaceae*_R-7_group significantly increased in group that treated with compound probiotic (*L. plantarum* and *B. velezensis*).

**Table 8:** Relative abundance of microorganisms at phylum and genus level (%).

Items	Groups				*p*-value
	CON	SP	DP1	DP2	
Phylum					
*Firmicutes*	70.95 ± 1.60	67.96 ± 2.30	65.94 ± 1.16	74.49 ± 4.82	0.237
*Bacteroidota*	24.79 ± 1.57	28.29 ± 2.79	30.47 ± 0.48	22.18 ± 3.98	0.185
*Proteobacteria*	1.32 ± 0.32	0.84 ± 0.14	0.87 ± 0.13	0.68 ± 0.10	0.192
*Desulfobacterota*	1.28 ± 0.11^a^	1.21 ± 0.06^a^	1.10 ± 0.06^a^	0.64 ± 0.20^b^	0.023
*Verrucomicrobiota*	0.46 ± 0.30	0.54 ± 0.41	0.37 ± 0.20	1.03 ± 0.95	0.843
*Fusobacteriota*	0.08 ± 0.01^a^	0.09 ± 0.02^a^	0.01 ± 0.00^b^	0.02 ± 0.01^b^	0.012
Genus					
*Muribaculaceae*	16.01 ± 2.85	16.79 ± 3.73	18.35 ± 2.07	12.83 ± 2.48	0.598
NK4A214_group	9.15 ± 0.74^a^	6.20 ± 0.28^b^	5.67 ± 0.54^b^	6.45 ± 0.69^b^	0.013
uncultured	8.60 ± 1.54	8.13 ± 0.29	7.87 ± 1.86	7.23 ± 0.89	0.896
*Lachnospiraceae_*NK4A136_group	6.93 ± 1.23	4.77 ± 0.48	5.48 ± 0.25	5.57 ± 0.87	0.347
*Christensenellaceae_*R-7_group	4.24 ± 0.53^b^	5.90 ± 0.45^ab^	4.30 ± 0.67^b^	7.22 ± 0.81^a^	0.029
*Ruminococcaceae*	1.85 ± 0.64	1.15 ± 0.55	1.48 ± 0.64	1.63 ± 1.06	0.922

The results are presented as the mean ± SEM. *n* = 3. The Kruskal–Wallis test was used. Different letters indicate significant differences (*p <* 0.05).

## 4 Discussion

It is well recognized that probiotics improve animal growth performance by fostering a healthy intestinal environment (26). Additionally, probiotics strengthen immune responses against viral or bacterial infections by promoting the dominance of beneficial microbiota and enhancing host immune function, ultimately improving overall animal health (6, 7). The immunity, antioxidant capacity and intestinal health maintaining during weaned period play a pivotal role in determining the growth and development of rabbits. Therefore, it holds significant production implications to investigate the impact of supplementary compound probiotics on these aspects of weaning rabbits. We hypothesize that compound probiotic might benefit the immunity, antioxidant capacity and health of weaned rabbits, thereby mitigate the substantial stress associated with weaning and ultimately leading to better production performance. To verify our hypothesis, the present study conducted a feeding trial with weaned rabbits to evaluate the impact of a separately or compound probiotic formulation on their growth performance, immune function, antioxidant capacity and intestinal barrier integrity.

In this study, dietary supplementation of probiotics (SP, DP1 and DP2 group) significantly enhanced the growth performance of weaned rabbits. Notably, supplementation with probiotic could significantly improve ADG and reduce the F/G, highlighting the potential of these probiotics to improve growth efficiency and lower feeding costs. We also found that the combined supplementation with *L. plantarum* QZF and *B. velezensis* BD significantly reduced meat shear force, indicating better tenderness and flavor. Our findings indicate that dietary supplementation with probiotics (especially compound probiotic with *L. plantarum* QZF and *B. velezensis* BD) can enhance growth performance, the feed conversion ratio and meat quality, which would provide favorable economic benefits in rabbit farming.

Notably, we found that dietary supplementation with *L. plantarum* QZF separately significantly increased BUN levels in weaned rabbits. We also found that the compound probiotic alleviates the elevation of BUN in the plasmic, particularly in the DP2 group. The dietary supplementation with *L. plantarum* QZF and *B. velezensis* BD can significantly reduce the elevation of plasmic BUN caused by *L. plantarum* (*p <* 0.05) and maintain it at a level similar to the control group. BUN is the end product of protein metabolism, mainly synthesized by the liver and excreted by the kidneys (27). The observed rise in BUN may reflect a shift in amino acid metabolism or an increase the burden on the liver or kidneys increase by *L. plantarum* QZF. This remains merely speculative and still need future studies to assess histological or functional biomarkers of the liver and kidney. These affords would help to more comprehensively determine the impact on amino-acid metabolism by probiotic such as *L. plantarum*. Besides, dietary supplementation with *L. plantarum* QZF and *C. fabianii* could elevate HDL-C levels in weaned rabbits, showing a trend toward increase. HDL-C is a type of lipoprotein in the blood, mainly functions to transport cholesterol from tissues to the liver for metabolism and has an anti-atherosclerotic effect (28). These results indicate that, compared to the separate supplementation of *L. plantarum* QZF, the compound probiotics are more effective in improving plasmic indicators and promoting cardiovascular health in animals.

Recent studies reported that compound probiotics exhibit favorable antioxidant effects in animals, such as ducks and broilers (29, 30). The enzymatic antioxidant defense system comprises superoxide dismutase (SOD), glutathione peroxidase (GSH-Px) and catalase (CAT) (31). SOD and CAT are widely recognized as two pivotal endogenous antioxidant enzymes that play an essential role in protecting against oxidative damage (32). In this study, we found that supplemented with *L. plantarum* QZF separately can increase the activity of CAT and SOD. However, when co-supplemented with *B. velezensis* BD or *C. fabianii* EMS, these positive effects in antioxidant capacity are weakened. We also found that compound probiotic (*L. plantarum* QZF and *C. fabianii* EMS) could significantly elevated the activity of GSH-Px. These complex effects may be attributed to microbial synergism or antagonism, where bacteria interactions could modulate their antioxidant-regulating effects in the host. Previous research has indicated that different bacterial strains (such as *Bacillus subtilis* and *Pseudomonas marginalis*) would compete for limited nutrients such as iron, sulfur and vitamins. Iron competition can affect bacterial production of SOD and CAT, thereby altering the antioxidant capacity of host (33). While individual probiotic may enhance antioxidant enzyme activity or regulate related genes, combinatorial administration might reduce antioxidant efficacy due to competition or metabolic interference (34). Recent studies suggested that the antioxidant activity of probiotics such as *Lactobacillus plantarum* or *Bacillus amyloliquefaciens* is likely mediated by activation of the Nrf2 signaling pathway (35, 36). However, we did not verify whether the Nrf2 pathway mediates the antioxidant effects of the compound probiotic in this study. Future validation of the involvement of the Nrf2 pathway might provide a targeted theoretical basis for optimizing the application of compound probiotics to enhance antioxidant capacity.

Studies have shown that probiotics regulate host immune function through multiple mechanisms, including inhibiting the adhesion and colonization of pathogens, regulating the function of immune cells, generating antibacterial substances and so on (37). Immunoglobulins (including IgA, IgM and IgG) play significant roles in humoral immunity to resist pathogens and prevent intestinal diseases (38). As shown in Figure 3, probiotic supplement in diet could significantly increase the plasmic levels of IgA in weaned rabbits compared to the control group. However, a compound probiotic supplement with *C. fabianii* EMS or *B. velezensis* BD will weaken the effect of *L. plantarum* QZF in enhancing plasmic antioxidant capacity, especially in IgG. This may be due to competitive colonization and competition for adhesion sites among the strains. In animal studies (such as pig), combined probiotics have exhibited colonization competition which diminishes the effects of individual strains (39). In this study, it is possible that *C. fabianii* or *B. velezensis* occupied a large number of adhesion sites, leading to a reduced colonization density of *L. plantarum*. As a result, its stimulation of host immune cells was weakened and decreased the level of immune factors in the plasmic.

The mucosal immune system plays a critical role in regulating immune homeostasis and preserving the integrity of the mucosal barrier (40). Immune factors in the intestine (such as sIgA, IL-1α and IL-2) play a key role in maintaining intestinal barrier function, regulating immune responses and inflammatory processes. (41). The upregulation of them indicated an enhancement of the intestinal immune system, which contributed to gut health and may prevent the occurrence of intestinal diseases. As shown in Figure 4, administration of compound probiotics (DP1 and DP2 group) could significantly increase the level of sIgA in the intestine. We also found that probiotic supplement would increase the levels of IL-1α and IL-2 in the jejunum. Our founding suggested that administration of suitable probiotic could modulate cytokine expression, enhance anti-inflammatory responses and promote immune homeostasis in weaned rabbits.

Probiotics could improve animal growth performance by maintaining a healthy intestinal environment (26, 42). The intestinal epithelial barrier, as the first line of host defences against pathogens and harmful substances, is crucial for maintaining the physiological metabolism of organism (43). The integrity of the intestinal mucosal structure is the foundation for maintaining the normal digestive function of the intestine. In our study, we found that compound probiotic has a significant positive effect on the intestinal morphology of duodenum. However, the addition of probiotics may have adverse effects on the intestinal morphology of the middle and posterior segments of the small intestine, particularly the jejunum. We also found dietary supplementation with probiotic exhibited higher level of amylase in duodenum compared to CON group (Table 5). This is similar to the effect of probiotics on intestinal morphology, which showing a positive effect in the duodenum. This may indicate that the effects of these probiotics on the gut are selective. We speculate that this may be due to differences in the colonization sites of these bacteria in the gut. Simply adding these probiotics without other nutritional strategies may not fully exert the potential of probiotics. Prebiotics have been shown to improve intestinal structure and alleviate intestinal inflammation (44, 45). Future research may consider the co-administration of probiotics and prebiotics to more effectively enhance animal gut health.

Probiotics could enhance fermentation of carbohydrates in intestine to produce short-chain fatty acids and thus inhibit the proliferation of pathogenic bacteria in the gut (46, 47). These short-chain fatty acids (such as acetic acid, propionic acid and butyric acid) can effectively lower the intestinal pH, inhibit the growth of pathogenic bacteria, maintain gut health and directly provide energy to intestinal cells. Our study indicates that the addition of a separately probiotic supplement with *L. plantarum* QZF in the diet results in significant lower pH in cecum content compared to the control group, while compound probiotic supplement weakened this effect. Notably, daily probiotic supplement would significantly increase the NH3-N content, which indicates enhanced microbial activity in the cecum. We also observed that the addition of probiotics increases the levels of acetic acid, propionic acid and butyric acid in the cecum, but the difference is not significant. However, compound probiotics significantly reduce the content of isovaleric acid in the cecum (*p <* 0.01). This may indicate that different probiotics have a selective effect towards fermentation substrates and the produced short-chain fatty acids, thereby exerting both positive and negative effects on cecal fermentation. Utilizing reasonable nutritional strategies, such as increasing the fiber content in diet, may enable compound probiotics to exert a more positive effect.

The intestinal microbiota and its metabolic products play crucial roles in maintaining gut homeostasis and regulating animal growth and development (48). To further investigate the impact of compound probiotic on the gut microbiota, we conducted 16S rRNA sequencing analysis on cecal contents. The result of Alpha and Beta diversity analysis indicated that dietary supplementation of these probiotics (*L. plantarum*, *B. velezensis* or *C. fabianii*) did not disrupt the gut microbial flora balance in healthy weaned rabbits. Notably, as shown in Figure 8 and Table 8, the abundance of *Fusobacteriota* was significantly decreased when supplied with compound probiotic (DP1 and DP2 groups). *Fusobacteriota* in the gut microbiota may participate in host health and disease and is associated with potential links to diseases such as colorectal cancer and inflammatory bowel (49, 50). Therefore, a decrease in the abundance of *Fusobacteriota* is often interpreted as a sign of reduced inflammatory risk and restored intestinal barrier function, indicating a positive effect of the compound probiotic on gut health. At genus level, the abundance of *Christensenellaceae*_R-7_group significantly increased in compound probiotic group treated with *L. plantarum* QZF and *B. velezensis* BD. As an important butyrate-producing bacterium in the gut, *Christensenellaceae*_R-7_group has a wide range of ecological and physiological functions, involving host metabolism, immune regulation and health maintenance (51, 52). Butyrate is the preferred energy source for colonic epithelial cells. Besides, Butyrate can activate the AMPK and PPARγ pathways, promote lipid oxidation and insulin sensitivity, thereby enhancing overall energy metabolism (53). Butyrate also plays a crucial regulatory role in the host immune system. It can suppress the excessive activation of dendritic cells, macrophages and neutrophils, activate immune mediators and alleviate intestinal inflammation (54). Therefore, supplementing with a compound probiotic to increase the abundance of butyrate-producing bacteria such as the *Christensenellaceae*_R-7_group can be regarded as an effective nutritional strategy for enhancing animal growth performance, reducing stress-induced inflammation and improving metabolic health. However, we also observed a significant decrease in the abundance of NK4A214_group in all probiotic-treated groups. As a bacterial group commonly found in the intestines of ruminants, NK4A214_group can degrade fiber and resistant starch, thus produce short-chain fatty acids such as acetate and butyrate (55, 56). We hypothesize that colonization of *L. plantarum* might compete with functionally similar gut microbes, thereby reshaping the microbial community. Therefore, the combined probiotics (*L. plantarum* QZF and *B. velezensis* BD) showed superiority over single strains in modulating the relative abundance of functional microbiota. This probiotic combination could also increased the relative abundance of *Christensenellaceae_*R-7_group. Overall, supplementation with compound probiotic can alter the abundance of specific gut microbiota to maintain a healthy microbial community. These results showed that the probiotic-supplemented treatment group exhibited differences in the abundance patterns of intestinal microbiota associated with intestinal barrier function and digestion. An increase in the abundance of possible beneficial bacteria may improve intestinal health and metabolic processes. However, since we used healthy rabbits in this study, the effects of compound probiotic on alleviating intestinal diseases and facilitating the restoration of disrupted gut microbiota balance were not demonstrated. Future studies will employ disease-challenge models (such as pathogen or stress-induced enteritis) to evaluate the efficacy of compound probiotic in maintaining intestinal health and microbiota balance.

In this study, we found that three dominant strains isolated from silage exhibited good probiotic properties. Specifically, *L. plantarum* QZF demonstrated enhanced antioxidant capacity in animals, as reflected by increased levels of SOD and CAT, as well as improved antioxidant activity and intestinal digestive enzyme activity. However, the application of *L. plantarum* alone significantly increased serum BUN levels, potentially increasing the metabolic burden on the liver and kidneys. This effect can be mitigated by combining *L. plantarum* with *B. velezensis* BD or *C. fabianii* EMS. The combination of *L. plantarum* and *B. velezensis* showed a more positive effect. However, the use of a compound probiotic weakened the effect of *L. plantarum* on enhancing serum antioxidant capacity. Additionally, the compound probiotic significantly improved the intestinal structure of duodenum, but had negative effects on jejunum and ileum. Overall, we believe that the compound probiotic offers more comprehensive and superior effects compared to single-strain formulations. However, the current application of compound probiotics still has some limitations. Therefore, further research is needed to explore strategies such as combining probiotics with prebiotics, adjusting feed nutritional composition and modifying fiber composition to maximize the potential of compound probiotics in animal production.

## 5 Conclusion

In summary, we isolated three strains of dominant bacteria (*Lactobacillus plantarum* QZF, *Bacillus velezensis* BD and *Cyberlindnera fabianii* EMS) from silage of *Pennisetum giganteum*. In this study, we demonstrated the beneficial impacts of these probiotics on modulating growth and development, antioxidant capacity, immunity, intestinal morphology and gut microbiota in weaned rabbits. The current works highlight the significant potential of compound probiotics in enhancing growth performance and intestinal health in animal production.

## Data Availability

The original contributions presented in the study are included in the article/Supplementary material, further inquiries can be directed to the corresponding author/s. The data of cecal microbiota analysis presented in the study are deposited in the SRA database, accession number PRJNA1345346.

## Footnotes

^1^ https://www.omicshare.com/tools

## Glossary

IBW: Initial body weight
FBW: Final body weight
ADFI: Average daily feed intake
ADG: Average daily gain
F/G: Feed to gain ratio
TP: Total protein
ALB: Albumin
TG: Triglyceride
TC: Total cholesterol
GLU: Glucose
BUN: Urea nitrogen
HDL-C: High-density lipoprotein
LDL-C: Low-density lipoprotein
SOD: Superoxide dismutase
MDA: Malondialdehyde
T-AOC: Total antioxidant capacity
CAT: Catalase
GSH-Px: Glutathione peroxidase
Ig: Immunoglobulin
IL: Interleukin
sIgA: Secretory immunoglobulin A
VH: Villus height
V/C: Villus height to crypt depth ratio
GC: Goblet cell
