# Supplementary material for: Effects of compound probiotics on growth performance, immunity, antioxidant capacity and gut microbiota in weaned rabbits
Source: Front Vet Sci. 2025 Nov 13;12:1714335. doi: 10.3389/fvets.2025.1714335 (PMC12661403; doi:10.3389/fvets.2025.1714335)
Supplement: Supplementary file 1 [file Table_1.docx]

Table S1. The results of Shapiro–Wilk test and Bartlett’s test in this study

| Items | Groups | | | | Shapiro–Wilk test | Bartlett’s test |
| --- | --- | --- | --- | --- | --- | --- |
|  | CON | SP | DP1 | DP2 |  |  |
| Growth performance | | | | |  |  |
| IBW (g) | 821.88 ± 28.49 | 775.00 ± 22.82 | 793.75 ± 27.72 | 831.25±28.09 | 0.04 | 0.69 |
| FBW (g) | 2283.13 ± 53.23^b^ | 2578.13 ± 61.57^a^ | 2550.00 ± 45.41^a^ | 2493.75 ± 42.05^a^ | 0.24 | 0.20 |
| ADFI (g) | 125.57 ± 2.93^b^ | 141.80 ± 3.39^a^ | 140.25 ± 2.50^a^ | 137.16 ± 2.31^a^ | 0.24 | 0.20 |
| ADG (g) | 24.36 ± 1.16^b^ | 30.05 ± 1.02^a^ | 29.27 ± 0.92^a^ | 27.71 ± 0.66^a^ | 0.41 | 0.26 |
| F/G | 5.25 ± 0.14^a^ | 4.75 ± 0.07^b^ | 4.83 ± 0.10^b^ | 4.97 ± 0.08^ab^ | 0.01 | 0.02 |
| Meat quality | | | | |  |  |
| pH24h | 6.46 ± 0.08 | 6.47 ± 0.05 | 6.58 ± 0.06 | 6.45 ± 0.01 | 0.08 | 0.19 |
| Drip loss (%) | 3.29 ± 0.91 | 2.32 ± 0.84 | 1.37 ± 0.44 | 2.42 ± 0.43 | 0.61 | 0.43 |
| Shear force (N) | 36.05 ± 5.02^a^ | 28.75 ± 5.45^ab^ | 36.2 ± 1.93^a^ | 20.96 ± 1.75^b^ | 0.89 | 0.35 |
| Plasmic metabolites | | | | |  |  |
| TP (g/L) | 53.90 ± 0.93 | 54.03 ± 1.52 | 54.00 ± 2.63 | 54.70 ± 2.36 | 0.79 | 0.19 |
| ALB (g/L) | 35.57 ± 0.64 | 36.63 ± 0.32 | 37.47 ± 1.68 | 35.40 ± 0.75 | 0.01 | 0.03 |
| TG (mmol/L) | 0.44 ± 0.07 | 0.59 ± 0.05 | 0.60 ± 0.06 | 0.55 ± 0.17 | 0.91 | 0.09 |
| TC (mmol/L) | 1.17 ± 0.21 | 1.55 ± 0.33 | 1.69 ± 0.14 | 1.70 ± 0.37 | 0.72 | 0.25 |
| GLU (mmol/L) | 6.07 ± 0.23 | 6.64 ± 0.34 | 7.00 ± 0.46 | 6.89 ± 0.24 | 0.64 | 0.49 |
| BUN (μg/mL) | 24.02 ± 3.02^b^ | 35.44 ± 2.81^a^ | 25.59 ± 3.12^ab^ | 19.15 ± 3.54^b^ | 1.00 | 0.32 |
| HDL-C (mmol/L) | 0.74 ± 0.07^b^ | 0.68 ± 0.02^b^ | 0.98 ± 0.07^a^ | 0.82 ± 0.07^ab^ | 0.08 | 0.02 |
| LDL-C (mmol/L) | 0.14 ± 0.02 | 0.35 ± 0.10 | 0.50 ± 0.09 | 0.65 ± 0.20 | 0.19 | 0.95 |
| Intestinal morphology | | | | |  |  |
| Duodenum | |  |  |  |  |  |
| Villus height (μm) | 938.43 ± 57.31^b^ | 925.79 ± 24.37^b^ | 1007.11 ± 40.37^ab^ | 1089.33 ± 35.16^a^ | 0.10 | 0.01 |
| Crypt depth (μm) | 96.15 ± 4.37^b^ | 91.05 ± 3.29^b^ | 113.47 ± 6.14^a^ | 113.75 ± 5.19^a^ | 0.14 | 0.09 |
| V/C | 10.11 ± 0.77 | 10.41 ± 0.55 | 9.44 ± 0.84 | 9.97 ± 0.72 | 0.41 | 0.43 |
| Goblet cell density (cells/mm) | 12.53 ± 0.74^a^ | 13.47 ± 0.77^a^ | 9.73 ± 0.35^b^ | 13.47 ± 0.93^a^ | 0.80 | 0.42 |
| Jejunum | |  |  |  |  |  |
| Villus height (μm) | 943.54 ± 84.64^a^ | 591.09 ± 17.23^b^ | 548.83 ± 22.15^b^ | 617.58 ± 23.44^b^ | 0.01 | 0.01 |
| Crypt depth (μm) | 120.09 ± 6.31^a^ | 103.76 ± 4.86^b^ | 86.05 ± 5.38^c^ | 105.81 ± 2.78^b^ | 0.21 | 0.19 |
| V/C | 7.99 ± 0.75^a^ | 5.80 ± 0.20b | 6.79 ± 0.58^ab^ | 5.88 ± 0.26^b^ | 0.01 | 0.01 |
| Goblet cell density (cells/mm) | 12.13 ± 2.37 | 7.47 ± 0.97 | 8.40 ± 0.69 | 8.13 ± 0.18 | 0.01 | 0.11 |
| Ileum | |  |  |  |  |  |
| Villus height (μm) | 617.77 ± 15.65 | 536.22 ± 37.27 | 597.83 ± 66.40 | 635.95 ± 26.04 | 0.09 | 0.01 |
| Crypt depth (μm) | 150.35 ± 7.88^a^ | 124.39 ± 4.93^b^ | 122.09 ± 6.25^b^ | 117.92 ± 13.50^b^ | 0.01 | 0.29 |
| V/C | 4.30 ± 0.30 | 4.37 ± 0.32 | 5.25 ± 0.77 | 5.95 ± 0.43 | 0.01 | 0.04 |
| Goblet cell density (cells/mm) | 17.67 ± 1.12^bc^ | 17.07 ± 0.33c | 19.80 ± 0.81^ab^ | 22.13 ± 0.47^a^ | 0.30 | 0.18 |
| Intestinal morphology | | | | |  |  |
| Duodenum | |  |  |  |  |  |
| Amylase (nmol/L) | 403.51 ± 3.91^c^ | 581.15 ± 12.25^a^ | 549.76 ± 8.64^b^ | 567.25 ± 6.97^ab^ | 0.01 | 0.52 |
| Cellulase (ng/mL) | 12.95 ± 0.92 | 18.16 ± 4.43 | 14.02 ± 2.98 | 16.37 ± 1.81 | 0.16 | 0.16 |
| Lipase (mg/mL) | 221.39 ± 12.91 | 225.43 ± 5.79 | 260.36 ± 24.93 | 217.92 ± 15.92 | 0.10 | 0.25 |
| Trypsin (μ/g) | 14.10 ± 1.70 | 12.11 ± 1.24 | 12.19 ± 3.48 | 11.56 ± 1.05 | 0.96 | 0.28 |
| Cecum | |  |  |  |  |  |
| Amylase (nmol/L) | 201.81 ± 1.02^b^ | 217.70 ± 1.29^a^ | 182.46 ± 1.55^c^ | 200.56 ± 1.27^b^ | 0.29 | 0.92 |
| Cellulase (ng/mL) | 20.90 ± 0.86 | 27.25 ± 7.00 | 25.31 ± 6.79 | 14.34 ± 1.58 | 0.04 | 0.09 |
| Lipase (mg/mL) | 239.99 ± 10.45 | 241.81 ± 9.58 | 225.31 ± 11.93 | 238.68 ± 4.50 | 0.76 | 0.40 |
| Trypsin (μ/g) | 12.43 ± 0.20 | 10.22 ± 1.43 | 10.69 ± 2.14 | 14.13 ± 2.40 | 0.79 | 0.11 |
| Cecal Fermentation | | | | |  |  |
| pH | 6.61 ± 0.04^a^ | 6.37 ± 0.04^b^ | 6.69 ± 0.08^a^ | 6.62 ± 0.02^a^ | 0.16 | 0.01 |
| NH_3_-N（µg/mL） | 1.03 ± 0.01^c^ | 1.25 ± 0.02^b^ | 1.23 ± 0.01^b^ | 1.34 ± 0.03^a^ | 0.15 | 0.26 |
| Acetic acid (μg/g) | 292.06 ± 14.61 | 415.08 ± 64.52 | 463.33 ± 139.45 | 443.05 ± 62.36 | 0.06 | 0.06 |
| Propionic acid (μg/g) | 100.01 ± 4.75 | 151.53 ± 34.20 | 139.37 ± 35.82 | 118.98 ± 3.04 | 0.01 | 0.07 |
| Butyric acid (μg/g) | 160.54 ± 16.30 | 248.19 ± 58.72 | 256.12 ± 104.77 | 259.89 ± 67.23 | 0.09 | 0.09 |
| Isobutyric acid (μg/g) | 19.36 ± 2.03 | 18.94 ± 1.28 | 13.74 ± 2.76 | 13.26 ± 1.10 | 0.93 | 0.21 |
| Isovaleric acid (μg/g) | 16.99 ± 1.81^a^ | 16.59 ± 0.83^a^ | 9.99 ± 1.49^b^ | 10.47 ± 0.83^b^ | 0.81 | 0.22 |

IBW, Initial body weight; FBW, final body weight; ADFI, average daily feed intake; ADG, average daily gain; F/G, feed/gain ratio; TP: Total protein; ALB: Albumin; TG: Triglyceride; TC: Total cholesterol; GLU: Glucose; BUN: Urea nitrogen; HDL-C: High-density lipoprotein; LDL-C: Low-density lipoprotein; V/C: Villus height/Crypt depth ratio. The results are presented as the mean ± SEM. The *p* value of Shapiro–Wilk test < 0.05 indicate the data do not meet normality; the *p* value of Shapiro–Wilk test < 0.05 indicate the data do not meet homogeneity of variance assumptions.

Table S2. Plasmic antioxidant capacity of weaned rabbits following probiotics supplementation.

| Items | Groups | | | | *p*-Value | Shapiro–Wilk test | Bartlett’s test |
| --- | --- | --- | --- | --- | --- | --- | --- |
|  | CON | SP | DP1 | DP2 |  |  |  |
| MDA (nmol/ml) | 0.71±0.16 | 0.84±0.10 | 0.82±0.15 | 0.69±0.09 | 0.416 | 0.46 | 0.70 |
| T-AOC (U/mL) * | 1.94±0.65 | 1.95±0.55 | 1.71±0.56 | 1.49±0.09 | 0.478 | 0.05 | 0.06 |
| SOD (U/mL) * | 64.54±24.77^b^ | 98.35±3.00^a^ | 62.32±1.99^b^ | 62.54±1.48^b^ | 0.048 | 0.19 | 0.01 |
| CAT (U/mL) * | 22.68±0.67^c^ | 46.25±0.93^a^ | 11.32±0.35^d^ | 25.41±0.68^b^ | 0.016 | 0.03 | 0.46 |
| GSH-H_2_O_2_ (nmol/mL) | 369.49±17.35^b^ | 364.41±20.09^b^ | 418.44±13.41^a^ | 396.57±16.36^ab^ | 0.015 | 0.98 | 0.91 |

SOD: Superoxide dismutase; CAT: Catalase; GSH-Px: Glutathione peroxidase; MDA: Malondialdehyde; T-AOC: Total antioxidant capacity. n = 3. * indicates that the data do not meet normality or the variances are not homogeneous., thus the Kruskal‑Wallis test was used. For others, ANOVA was used. The results are presented as the mean ± SEM. Different letters indicate significant differences (*p* < 0.05).

| Items | Groups | | | | *p*-Value | Shapiro–Wilk test | Bartlett’s test |
| --- | --- | --- | --- | --- | --- | --- | --- |
|  | CON | SP | DP1 | DP2 |  |  |  |
| IgA (ug/mL) | 194.19±7.47^c^ | 222.93±8.12^b^ | 253.06±4.97^a^ | 222.38±10.81^b^ | 0.001 | 0.86 | 0.64 |
| IgM (ug/mL) | 442.22±22.19 | 494.74±17.19 | 487.61±44.25 | 460.34±11.76 | 0.136 | 0.89 | 0.35 |
| IgG (g/L) | 10.35±0.70^b^ | 12.49±1.29^a^ | 11.97±0.49^ab^ | 11.47±0.80^ab^ | 0.048 | 0.80 | 0.21 |

Table S3. The immune factor levels in the plasma.

IgG: Immunoglobulin G; IgA: Immunoglobulin A; IgM: Immunoglobulin M. The results are presented as the mean ± SEM. ANOVA was used. Different letters indicate significant differences (*p* < 0.05). n = 3.

Table S4. The immune factor levels in the intestinal.

| Items | Groups | | | | | *p-*Value | Shapiro–Wilk test | Bartlett’s test |
| --- | --- | --- | --- | --- | --- | --- | --- | --- |
|  |  | CON | SP | DP1 | DP2 |  |  |  |
| sIgA (ug/g) | Duodenum * | 8.52±0.15^b^ | 8.80±0.44^b^ | 10.80±0.40^a^ | 10.97±0.55^a^ | 0.034 | 0.05 | 0.25 |
|  | Jejunum * | 10.17±0.14^b^ | 10.25±0.46^b^ | 13.27±0.36^a^ | 13.22±0.54^a^ | 0.037 | 0.02 | 0.29 |
|  | Ileum * | 9.90±0.41^b^ | 9.78±0.35^b^ | 13.08±0.25^a^ | 12.78±0.59^a^ | 0.038 | 0.02 | 0.60 |
|  | Colon * | 9.02±0.12^b^ | 8.66±0.44^b^ | 12.03±0.15^a^ | 11.94±0.62^a^ | 0.036 | 0.02 | 0.13 |
| IL-1α (ug/g) | Duodenum | 17.27±0.43^c^ | 21.88±0.39^a^ | 21.47±0.54^a^ | 19.20±0.42^b^ | 0.001 | 0.12 | 0.86 |
|  | Jejunum * | 23.05±0.44^b^ | 28.54±1.06^a^ | 27.46±0.87^a^ | 27.59±0.60^a^ | 0.046 | 0.04 | 0.57 |
|  | Ileum | 26.10±0.75^c^ | 34.11±0.61^a^ | 31.44±0.21^b^ | 31.47±0.87^b^ | 0.001 | 0.07 | 0.33 |
| IL-2 (ug/g) | Duodenum * | 54.36±0.76^c^ | 69.00±1.38^a^ | 67.07±2.10^ab^ | 64.61±1.09^b^ | 0.025 | 0.02 | 0.38 |
|  | Jejunum | 71.77±1.63^d^ | 82.32±0.51^a^ | 79.29±1.42^b^ | 76.62±0.81^c^ | 0.001 | 0.53 | 0.30 |
|  | Ileum * | 83.59±1.43^b^ | 93.33±0.55^a^ | 93.63±1.95^a^ | 81.90±2.16^b^ | 0.034 | 0.05 | 0.26 |

sIgA: Secretory immunoglobulin A; IL-1α: Interleukin 1α; IL-2: Interleukin 2. The results are presented as the mean ± SEM. n = 3. * indicates that the data do not meet normality or the variances are not homogeneous., thus the Kruskal‑Wallis test was used. For others, ANOVA was used. Different letters indicate significant differences (*p* < 0.05).
